# Congestion Control for a Fair Packet Delivery in WSN: From a Complex System Perspective

**DOI:** 10.1155/2014/381305

**Published:** 2014-08-10

**Authors:** Daniela Aguirre-Guerrero, Ricardo Marcelín-Jiménez, Enrique Rodriguez-Colina, Michael Pascoe-Chalke

**Affiliations:** Department of Electrical Engineering, Metropolitan Autonomous University (UAM), 09340 Mexico, DF, Mexico

## Abstract

In this work, we propose that packets travelling across a wireless sensor network (WSN) can be seen as the active agents that make up a complex system, just like a bird flock or a fish school, for instance. From this perspective, the tools and models that have been developed to study this kind of systems have been applied. This is in order to create a distributed congestion control based on a set of simple rules programmed at the nodes of the WSN. Our results show that it is possible to adapt the carried traffic to the network capacity, even under stressing conditions. Also, the network performance shows a smooth degradation when the traffic goes beyond a threshold which is settled by the proposed self-organized control. In contrast, without any control, the network collapses before this threshold. The use of the proposed solution provides an effective strategy to address some of the common problems found in WSN deployment by providing a fair packet delivery. In addition, the network congestion is mitigated using adaptive traffic mechanisms based on a satisfaction parameter assessed by each packet which has impact on the global satisfaction of the traffic carried by the WSN.

## 1. Introduction

A wireless sensor network (WSN) consists of a large number of nodes, which have sensing, processing, and communicating capabilities. Nodes in a WSN do not only monitor their environment, but they also forward and route data packets to one or more appointed* sink* nodes. It is known that there are open issues that limit the practical adoption of WSNs [1]. These limitations come from the fact that each individual wireless node in a WSN has reduced processing capabilities as well as limited energy budget.

Network congestion occurs when the system is close to its carrying capacity and an increase on the incoming traffic, or even a burst, may overload node buffers with a potential domino effect that may turn into a disaster. On these conditions, and unless a congestion control mechanism is used, the number of packets arriving to their final end falls abruptly. In contrast, a network under congestion control keeps close to an ideal response. That is, the outgoing traffic equals the network carrying capacity. Additionally, for WSN applications, congestion control mechanisms should also consider the side effects of wireless transmissions, such as interference and power loss.

In recent years, a set of models and tools initially developed by the physics community have shown their pertinence to address problems from other domains, such as biology and economics, among many others. The objects studied from this emerging body of knowledge are generally called complex systems. Mitchell [2] describes complex systems as* “… made up from a massive network of interacting components, without a central control, that follow a simple set of operational rules and are able to achieve an elaborated collective behavior, thanks to sophisticated information processing techniques and adaptation, based on learning or evolution.”* We can immediately identify many systems that fit into this definition, for example, ant colonies, bird flocks, fish schools, the international economy, the city vehicle traffic, and so forth. We know that none of them have a central control. We say that the interactions of components occur in a microscopic level. Nevertheless, in a macroscopic level, all of them show an emergent behavior which adapts to changing conditions over the time.

In this work, we agree with other authors that a WSN can be described as a complex system. The novelty of our approach is based on the fact that packets are regarded as the key entities that shape the overall system behavior. From our perspective, there are two types of interactions, among packets and packets with nodes. At node level, packets compete for network resources (buffers and links) on their way to the sink node(s). Based on these local interactions, each node is programmed to take decisions about routing, medium contention, and packet dropping. As an emergent global behavior, it is shown that the network achieves a self-adaptive mechanism that matches the traffic with the network capacity. An underlying assumption of our work is that, as it was stated by the economist Adam Smith, there exists an “invisible hand” that guides individual packets to benefit the system through the pursuit of their private interests.

Our experimental results show that the proposed solution may provide an effective strategy to address some of the common problems found in WSN deployment, such as fair packet delivery, adaptive routing, energy consumption, and congestion control.

The rest of this work is comprised of the following sections.  Section 2 summarizes relevant work found in the literature related to a variety of control mechanisms for wireless sensor networks.  Section 3 describes the theoretical framework on which the proposed method is based on.  Section 4 presents the experimental platform and a diversity of methods used to evaluate the performance of the proposed scheme. We describe and analyze in Section 5 the results obtained by simulations and we also discuss some relevant aspects that should be taken into consideration during the scheme implementation. Finally, Section 6 provides some concluding remarks.

## 2. Related Work

In this work, we introduce a model that comprises the idea of self-organized agents that can modulate a global behavior which, in our case of study, turns into the congestion control of a wireless sensor network. Our approach is strongly inspired by agent-directed simulations [3]. To the best of our knowledge, this approach is barely found in the literature of WSN, with a few related alternatives, such as [4], where authors propose the use of the complex system theory to deal with WSN. In our work we proposed a similar analogy but additionally we show results for specific applications and scenarios as well as a novel mechanism for the traffic control based on the packet satisfaction where the packet is regarded as the agent of the complex system.

In [5], a methodology for designing a self-organized WSN is presented. The authors suggest that cooperation between nodes is needed in order to accomplish more complex tasks for WSNs. In addition, they mentioned that a promising approach of how to cope with this is the use of the emergent self-organization. The proposed emergent self-organizing system aims to achieve improved scalability by the simplicity of its elements and their interactions. We agreed with the authors in [5] with the macroscopic and microscopic approach; however, we proposed a subtle difference from that work in the distributed coordination mechanism, because they use an explicit feedback loop mechanism but we proposed the packet interactions with the nodes as main distributed coordination mechanism without a specific control mechanism based on positive and negative feedback loops. In our proposed mechanism the packet satisfaction is the main parameter to adjust the traffic flow without the need of an explicit feedback loop between nodes.

A more comprehensive approach is presented in [6], which introduces an agent-based simulation framework. This work focuses on modeling of sensed variables of the environment rather than a WSN itself. The work presented in [7, 8] also considers packets as active entities of a complex system. From an agent-based approach, an improvement on directed diffusion routing protocol is presented in [7], whereas in [8] a routing protocol inspired on bird flocking is proposed.

In [9], a congestion control is presented for sensor networks with an algorithm independent of the topology and any addressing scheme. Then, the authors emphasize that the absence of state information per node in the network becomes a critical issue if a congestion control algorithm is required. Thus, a common approach is to provide a solution based on biological mechanisms for self-organization and complex systems [9–11]. In our proposed model shown in this work, an important viewpoint introduced is that data packets circulating over the network can be described as independent entities that are shaping an emergent global process. The interaction rules that we introduce are settled on a local and short-termed level. Nevertheless, the resulting emerging properties are studied from a global perspective.

Several publications have paid attention to the fact that the WSNs are comprised of resource-constrained devices [11–13]. Therefore, their design is oriented to minimize efforts without compromising the task's integrity and the gathering of the vast information required for a huge and unknown deployed area.

In recent years, many protocols and implementations have been developed for congestion control in wireless sensor networks. The most popular approach is based on traditional methodologies and protocols recognized from the Internet. For instance, several of them have studied the congestion problem from the transport and link layers perspective. However, the possibility of developing congestion control of a network during the packet trip from source to destination has not been explored in depth.

The authors in [14] propose a multiagent system with the ability of its agents to find alternative paths for energy efficient data dissemination. This approach is mainly used for routing. In [15] the network is able to gather information using the multiagent approach, in order to take further decisions. In contrast, we propose a simple mechanism to detect changes in the network conditions without considering anything but the degree of satisfaction of the individual packets. Satisfaction is a measure that estimates whether a packet has the possibility to reach its final destination within a bounded time.

## 3. Theoretical Framework

According to [16], a complex system is made up from a set of entities, called agents, which are deployed and act over a given environment. Agents have goals and show a particular behavior oriented to achieve such goals. From this perspective, it is important to recognize not only the acting elements of a system, but also the interactions of the agents with their environment (agent-to-environment), as well as the interactions among themselves (agent-to-agent).

In this work, we state that the traffic on a WSN, under stressing conditions, can be studied from a complex system perspective. Data packets are regarded as agents, whose goal is to reach a given sink, with minimum delay. In this case, the interactions between the packets and their environment can be described in terms of the network resources used by packets travelling to the sink, that is, links and buffers. Notice, for instance, that the conditions of a wireless link may induce undesired effects, such as noise and power loss, which have impact on the packet reception. Also, the size of the buffer limits the number of packets that can be temporarily stored in a given node. The interactions among packets arise from the fact that they compete for the network resources and this condition may cause network congestion, whose effects can be detected either at the link level or at the node level. In the former, it produces packets collisions. In the latter, it increases the delivery time, and under very stressing conditions it produces packet losses.

### 3.1. Taking the Pulse of the System

In order to evaluate the impact of the aforementioned interactions, we propose two measures that allow us to study the network traffic from a microscopic view. The first one estimates the fulfillment of a packet goal. The second one estimates the interference produced by a packet while interacting with other packets. Besides, we developed a macroscopic measure to estimate the overall network performance.

We can assume that each node of the network takes a snapshot of its current state, according to some schedule, including its buffer occupancy. Based on this state, each node calculates the following measures.

#### 3.1.1. The Individual Packet Satisfaction

**Figure 1:**
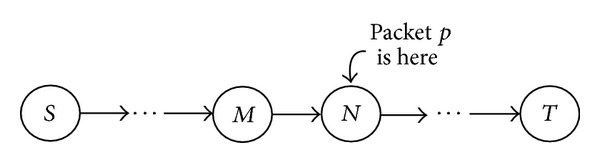
Packet *p* travelling from source node (*S*) to sink node (*T*).

Let *p* be a packet travelling over a WSN, from a source *S* to a target sink *T*. Let us also assume that *p* has been temporarily stored in node *N*, which is the *k*-th stage on its route, after being forwarded from node *M* (see Figure 1). The packet satisfaction of *p* is assessed by the variable *σ*_*p*,*N*_ ∈ [0,1] and is calculated at each node it traverses (visits), including *S*. Accordingly, the satisfaction of *p* at node *N* is determined by the following expression:

(1) $$\begin{array}{c} \sigma_{p,N}=\sigma_{p,M}+\frac{\left(1-\Delta t/\Delta t_{\max}\right)-\sigma_{p,M}}{k}, \end{array}$$

where Δ*t* is the time that *p* has been stored in the buffer at node *N*, while Δ*t*_max⁡_ is the maximum time that *p* is allowed to remain in a buffer before it has to be discarded; then 0 < Δ*t* ≤ Δ*t*_max⁡_. As we just mentioned, (1) applies to each node on the route of *p*, including node *S*. For the initial case at the departing node, *k* = 1 and *σ*_*p*,*M*_ = 0.

The first step for *N* to calculate *σ*_*p*,*N*_ is to determine the normalized delay experienced by *p* while buffered at *N*. This delay is given as follows:

(2) $$\begin{array}{c} \left|\Delta t\right|=\left(1-\frac{\Delta t}{\Delta t_{\max}}\right).\; \end{array}$$

The second step consists in evaluating the change on the packet satisfaction (Δ*σ*_*p*,*N*_), which is given by the contribution of *N* to the normalized delay of *p*; that is,

(3) $$\begin{array}{c} {\Delta \sigma }_{p,N}=\frac{\left|\Delta t\right|-\sigma_{p,M}}{k}. \end{array}$$

Therefore, (1) can be written in terms of Δ*σ*_*p*,*N*_, as in the following:

(4) $$\begin{array}{c} \sigma_{p,N}=\sigma_{p,M}+\Delta \sigma_{p,N}. \end{array}$$

Notice that *σ*_*p*,*N*_ can be understood as a function that updates the accumulated delay of *p* on its journey from source to sink.

Each node on the route traveled by packet *p* must be able to label the packet with its corresponding time of arrival in order to estimate Δ*t*. As for Δ*t*_max⁡_, this is a parameter that features each node, and it is oriented to shape desired properties of the traffic that will be carried by the network. For instance, Δ*t*_max⁡_ can be settled according to the distance from a given node to the sink, in such a way that a node which is near to the sink will have a value greater than those nodes that are rather away from the sink. Thus, we foresee that packets travelling long routes do not accumulate an excessive delay at the beginning of their journey, while a longer delay is expected at nodes that are near the sink.

#### 3.1.2. Interference among Packets

Let *N* be a node with a buffer that is temporarily occupied by a group of *F* packets, called* flock*, which includes packet *p*. The interference (*ϕ*_*p*_) assesses the way that *p* affects the satisfaction of the flock it belongs to according to the following expression:

(5) $$\begin{array}{c} \phi_{p}=-\Delta \sigma_{p,N}-\frac{1}{F-1}\sum_{i\neq p}\Delta \sigma_{i,N}. \end{array}$$

Equation (5) shows an algebraic addition with two parts. The first part corresponds to the satisfaction change experienced by packet *p* at the current node (−Δ*σ*_*p*,*N*_). The second part is the average change experienced by the rest of the packets that share the current buffer with *p*, that is, (−(1/(*F* − 1))∑_*i*≠*p*_Δ*σ*_*i*,*N*_).

If the current change of the normalized packet delay is greater than its accumulated delay, then it results in a negative change, which means that the packet satisfaction has been reduced at the current node. Let us recall that the satisfaction is a number between 0 and 1, where 1 represents the maximum satisfaction and 0 a null satisfaction.

Now, let us assume that (5) produces a positive interference, called* friction*, which is an undesirable condition. This implies the following.

1. Both of its parts are negative. In this case, we say that both sides have lost satisfaction.
2. The change in the satisfaction of *p* is negative, while the average change in the rest of the packets is positive. Nevertheless, the absolute value of the first part is greater than the second. In this case we say that the loss of satisfaction on one side is not compensated by the increase on the other side.
3. The change in the satisfaction of *p* is positive, while the average change in the rest of the packets is negative. Nevertheless, the value of the second part is greater than the first. We also say that the loss of satisfaction on one side is not compensated by the increase on the other side.

Now, let us assume that (5) produces a negative interference, called* synergy*, which is a desirable condition. This implies the following.

1. Both of its parts are positive. In this case, we say that both sides have gained satisfaction.
2. The change in the satisfaction of *p* is positive, while the average change in the rest of the packets is negative. Nevertheless, the absolute value of the first part is greater than the second. In this case, we say that the gain of satisfaction on one side does not strongly affect the loss on the other side.
3. The change in the satisfaction of *p* is negative, while the average change in the rest of the packets is positive. Nevertheless, the value of the second part is greater than the first. We also say that a gain of satisfaction on one side does not strongly affect the other side loss.

Therefore, there are three possible interactions between packet *p* and the flock it belongs to.

If *ϕ*_*p*_ > 0, then the packet *p* introduces* friction* on the flock, which is an undesirable condition.

If *ϕ*_*p*_ < 0, then packet *p* fosters* synergy*, which is a desirable condition.

If *ϕ*_*p*_ = 0, then the change on packet satisfaction cancels the change on the satisfaction of the others. In this case we say that *p* has a* neutral interaction* with the flock.

#### 3.1.3. Overall System Performance or a Macroscopic View of the Network

Let us suppose now that we are able to synchronize, at time *t*, the snapshots taken by the overall set of nodes that make up the WSN and gather these pictures to elaborate a global diagnostic of the system. We assume that, at this point in time, there are *n* packets stored at different places. For a packet, with identifier *i* = 1, …, *n*, we say that this packet is stored at a given node *N*(*i*) and, at time *t*, this node takes its local snapshot. The network satisfaction is estimated according to the following expression:

(6) $$\begin{array}{c} \sigma_{\text{WSN}}\left(t\right)=\frac{1}{n}\overset{n}{\underset{i=1}{\sum }}\sigma_{i,N(i)}. \end{array}$$

This expression means that, at the time of the snapshot, we evaluate the average satisfaction of the packets traveling across the network.

### 3.2. Congestion Control for WSN under Heavy Traffic Load

Now, we present a new congestion control based on the aforementioned framework. Our proposal is targeted to maximize the network satisfaction *σ*_WSN_(*t*).

The solution consists of four stages: (1) initialization, (2) processing, (3) routing, and (4) transmission. Initialization is executed only once in order to settle down the working parameters of each active node. Processing takes place each time a new packet is received at the node in order to estimate its microscopic measurements. Based on these estimates, and during the routing stage, the node decides whether to forward the packet immediately, store it at its local buffer, or drop it. Finally, if transmission applies, the node invokes a CSMA MAC protocol, which includes a modified version of an adaptive back-off interval, to forward the packet to the next node.

#### 3.2.1. Initialization

During initialization, the sink node grows a spanning tree rooted at this node [17]. We say that each node lying on the tree has a corresponding level (*l*). This level is related to the minimum number of hops from a given node to the sink. The root has a level 0; the children of the root have a level 1, and each node has the level of its father plus one. Each node knows its level as well as the level of its neighbors.

We consider that the workload of a given node depends on its distance to the sink. This means that the nodes in the vicinity of the sink concentrate on a higher load and therefore, they are expected to show a higher buffer occupancy compared to those nodes in the borders of the network. As a consequence, we also consider that those packets arriving at the last stages of their trip will experience longer delay. From this perspective, we assume that the processing of a packet must take into account the relative position of the node that is traversing, on its way to the sink. It is important to recall that the “goal” of an individual packet is to arrive to its final destination with the higher satisfaction, that is, the smallest accumulated delay. In contrast, the goal of the network, from a macroscopic view, is to keep congestion under control. Also, it is important to realize that the accumulated delay of a packet is strongly related to the flock size defined at the node where it is temporarily stored.

Assuming that all nodes have a uniform buffer size, given by *b*, (measured in bits), and in order to address the aforementioned considerations, the flock size must be related to the position of the node which allocates this set of packets. In general terms, a small flock introduces smaller delays than a bigger flock. If we accept that a packet should accumulate the least possible delays at the beginning of its journey and that it should be prepared to tolerate longer delays at the end, we conclude that the flock size must be a fraction of the buffer size, reflecting the distance of the node to the final target. To estimate this distance we may use the level of the node, according to the spanning tree that the sink has built over the underlying graph. Nevertheless we simplified this approach and decided to classify the network in three zones only: distant, intermediate, and nearby, all measured with respect to the sink node. For this purpose, we can think of the network as divided by three concentric “circles” drawn around the sink. These circles define the boundaries of such zones.

Let *L* be the highest level that can be achieved by a node in the network, that is, the maximum hop distance to the sink node. Then, we divide the spanned region into three zones according to *L*. A node having level *l* is considered to lay on zone 1, 2, or 3, according to the following expression:

(7) $$\begin{array}{c} \text{Zone}=\{\begin{array}{cc} 1, & 0<l<\left\lfloor \frac{L}{3}\right\rfloor ,\\ 2, & \left\lfloor \frac{L}{3}\right\rfloor \leq l<\left\lfloor \frac{2L}{3}\right\rfloor ,\\ 3, & \left\lfloor \frac{2L}{3}\right\rfloor \leq l. \end{array} \end{array}$$

As we stated before, packets are gathered in groups, called* flocks*. The size of a flock (*f*), measured in bits, depends on the level of the node where this flock is currently stored. Notice that *F* represents the number of packets that make up a flock, while *f* represents the overall “weight” of this group. Consider

(8) $$\begin{array}{c} f=\{\begin{array}{cc} b, & \forall l\in \text{Zone}\;1,\\ \left\lceil \frac{2b}{3}\right\rceil , & \forall l\in \text{Zone}\;2,\\ \left\lceil \frac{b}{3}\right\rceil , & \forall l\in \text{Zone}\;3, \end{array} \end{array}$$

where *b* is the buffer size, in bits. Let us recall that we are assuming that all nodes have the same buffer size.

Equation (8) implies that, depending on the zone where a given flock is travelling, the flock occupies one-third, two-thirds, or the overall buffer size, corresponding to zone 3, 2, or 1, respectively. In turn, this means that as the flocks get closer to the sink they can grow and, as a consequence, the may experience longer delays.

Each node configures its local time-to-live (TTL) parameter, which indicates the maximum number of hops that a packet may travel to reach the sink. A node on the role of source will use this parameter to stamp each issued packet. As it can be deduced, the particular value of the TTL depends on the zone where the corresponding node lies. Consider

(9) $$\begin{array}{c} \text{TTL}=\left\lceil \frac{4l}{3}\right\rceil . \end{array}$$

Let us notice that a long TTL value may foster the existence of packets that travel around the sink without finishing their trip but interfere with the rest of the packets and reinforce congestion. Therefore, TTL should be settled in order to give a packet a rather small chance to evade a troubled area before it is discarded.

Notice that the minimum number of hops that packets need to reach the sink is equal to level *l* of its source node. However, packets are settled with extra hops to dodge congestion zones. In order to finish the initialization stage, each node settles a value Δ*t*_max⁡_ that corresponds to the maximum time a packet may be stored in its buffer. The value of Δ*t*_max⁡_ depends on the zone where the node belongs to, according to the following expression:

(10) $$\begin{array}{c} \Delta t_{\max}\;=\frac{f-w}{v}, \end{array}$$

where *w* is the packet length, in bits, such that *w* < *f* and *v* is the local transmission rate, in bps.

Let us imagine that a packet arrives to a given node and it happens to be the last that completes a whole flock. In other words, regarded as a queue, this is the missing client that starts the forwarding service. How long will it wait to continue its trip? Well, it has to wait for the rest of its flock to be dispatched before it takes its turn.

From the above expressions, we observe that most of the working conditions of a node strongly depend on its distance to the sink, which is roughly estimated by its corresponding zone. In this way, we not only foresee that the nodes on the vicinity of the sink are able to tolerate high buffer occupancy, during harsh conditions, but also foster that packets coming from the farthest places do not accumulate long delays at the beginning of their trip.

#### 3.2.2. Processing

When a source node *S* issues a new packet *p*, it labels such packet with an initial value *σ*_*p*,*S*_. Then, each node *N* visited by *p* updates this value according to the following steps.

Before storing *p* in its buffer, *N* records the value *σ*_*p*,*M*_ that *p* is carrying in its header and reduces the TTL field of *p* by 1. If TTL reaches 0, it simply drops the packet, otherwise it stores *p*.

When *N* has gathered a flock, it retrieves each packet *p* from its buffer and updates the header of *p* with a new value *σ*_*p*,*N*_. Also, *N* calculates the interference caused by *p* on the flock it belongs to, according to the expressions (4) and (5), respectively.

The interaction of *p* with the rest of the flock may produce friction or synergy or may be neutral depending on the following cases.

**Case A.**

Friction (*ϕ*_*p*_ > 0).

**Case B.**

Synergy (*ϕ*_*p*_ < 0).

**Case C.**

Neutral (*ϕ*_*p*_ = 0).

Concurrently with these steps, each node is regularly scanning its buffer load. If it finds a stored packet which is about to reach its maximum storage time (Δ*t*_max⁡_), it retrieves this packet immediately and places it on the routing stage. Otherwise, the node waits to gather a whole flock to enter into this stage.

#### 3.2.3. Routing

Based on the packet interference, this phase applies an adaptive mechanism that may produce either a vertical or a horizontal routing decision. The vertical and horizontal routing decisions depend on the distance from the sink to the node distributing the traffic. The routing decision is horizontal when a node forwards traffic to other nodes with equal level or hop distance. The routing decision is vertical when nodes forward packets to other nodes that are closer to the sink; it means nodes with lower level.

Once the node has updated the value *σ*_*p*,*N*_, it checks whether there is a packet with a value Δ*σ*_*p*,*N*_ < 0. If there is not a single packet in this condition, the flock goes to the transmission stage to be forwarded in a vertical routing. Otherwise, for each packet with Δ*σ*_*p*,*N*_ > 0, it verifies if packet *p* introduces friction; that is, Δ*σ*_*p*,*N*_ > |∑_*i*≠*p*_(Δ*σ*_*i*,*N*_/(*F* − 1))|. The packets that do not meet this condition, along with those packets such that Δ*σ*_*p*,*N*_ < 0, go to the transmission stage to be forwarded in a vertical routing. The remaining packets are forwarded in a horizontal routing.

#### 3.2.4. Transmission

In this stage we assume the existence of a medium access control based on CSMA. Nevertheless, this scheme includes an adaptive back-off interval that depends on the zone where the corresponding node has been deployed. The node in charge forwards a packet according to the following rules.

1. The node senses the medium to find out whether it is busy or idle.
  - If the medium is busy, it programs a back-off timer and goes back to step (1), when the timer expires.
  - Otherwise, the node sends a “request-to-send” packet (RTS) to the immediate target node(s); this packet includes the number of data packets which is about to forward.
2. A node that receives the RTS packet answers with a “clear-to-send” (CTS) packet including the number of data packets which is able to store in its buffer.
3. Upon receiving the answers, the issuing node forwards the rest of the flock to as many nodes as possible. Transmission starts with those packets having the minimum Δ*σ*_*p*,*N*_.
4. When the node has an empty buffer, it measures its remaining battery, and in case it has less than 10% of its initial charge, it broadcasts a “farewell” message to all of its neighbors to let them know that it is about to “die.”
5. A node that receives this message eliminates the issuer from its routing tables.

## 4. The Experimental Platform and the Methods

Simulation tools are the best suited resources when analytic methods are unable to provide accurate solutions, or when direct experiments are not feasible. Both conditions can be met in WSN. Due to the amount of interacting entities, analytic tools can hardly assess the parameters that feature the system's performance. In addition, deploying an experimental settlement can be expensive and the obtained results may not be considered for a more general framework. Therefore, many specialists on WSN consider simulation tools as the departing point for experiments of network operations.

We developed a simulation model oriented to test and evaluate various congestion control schemes and routing mechanisms. The model is based on the description of the network traffic from the agent-based perspective. It is important to underline that we present a model that can be implemented using any tool supporting an agent-based specification, as well as discrete events. Our particular implementation is based on NetLogo [18], because it is a well-known tool which supports the development of a model where agents interact at discrete points in time and space.

### 4.1. An Agent-Based Model

Agent-directed simulation [3] is used to emulate a complex system behavior, such as the system analyzed in this work. The key premise that guides this analysis is that friction reduction between agents benefits the system satisfaction, thus increasing performance of the WSN. Maximizing *σ*_WSN_(*t*) can be accomplished by limiting the action of those agents that reduce the value *σ*_*p*,*N*_ of others, while preserving functionalities and fostering synergy among them.

As agents may present conflicting goals, their behavior is limited or regulated by means of mediators. These entities are in charge of minimizing conflicts, interferences, and frictions in order to maximize cooperation and synergy. In our particular case, nodes play the role of mediators.  Table 1 shows the correspondence between concepts used in WSN and complex systems. These equivalent concepts are the key to the construction of the proposed experimental platform.

### 4.2. A WSN Simulation Tool

The tool that we have devised comprises the following set of modules:

- network deployment and properties,
- packet generation,
- routing,
- congestion control,
- wireless effects,
- energy consumption,
- performance evaluation.

#### 4.2.1. Network Deployment

This module fixes the space and time properties of the supported WSN model. It describes the extension of the area, spanned by the WSN, as well as its shape, for example, square, circle, or irregular. Also, it settles the total simulation time as well as the length of the window that measures the value of *σ*_WSN_(*t*). It is known that many control mechanisms strongly depend on the time scale chosen to introduce adaptive decisions, but also the effectiveness of these mechanisms becomes evident depending on the time scale chosen to evaluate the system's performance: “an apparently bad decision in the short term may turn into a good decision in the long term.”

In our model we consider 3 types of nodes.

- *Sink:* it is the final destination of the packets carried by the network. Therefore, the outgoing traffic, that is, throughput, is measured considering the number of packets delivered at this point.
- *Source:* these nodes generate the incoming traffic; that is, the packets that are carried by the network towards the sink. If necessary, these nodes may also develop forwarding operations.
- *Forwarding:* these nodes are in charge of routing the packets on their way to the sink.

The number of deployed nodes is defined by the user, who also defines the number of source nodes. The sink has a random location, as the rest of the deployed nodes.

The average node degree defines the average number of connections each node has. In WSN, nodes' degree can be featured as a random variable that follows a probability distribution function (PDF). In this particular case, we generate a random number using a normal distribution and then we round it to the closest integer. According to this characteristic, each node is connected with those nodes within its propagation radio. The global effect of these local connections is the resulting graph that models the underlying communications network.

Due to the fact that coverage directly depends on the transmission power, coverage also affects the nodes' lifetime. A wider coverage area means that the corresponding node is running out of its energy more rapidly. Coverage is also considered a random variable that follows a normal distribution.

Other parameters that are settled within this module are buffer size, transmission rate, packet size, error rate, end-to-end delay, and energy consumption per packet which is related to coverage.

#### 4.2.2. Packet Generation

The traffic scenarios are simulated by traffic generated at the source nodes. This has the possibility of issuing a given number of packets per unit time, according to any of the following options:

- generating a random number using a normal PDF and then it is rounded to the closest integer,
- generating a random number using a Poisson PDF,
- a continuous generation rate,
- an augmenting rate that grows “*x*” packets per unit time, every fixed time step.

#### 4.2.3. Routing

The routes from all sources to the sink node are established once at the beginning of each simulation, using a well-known distributed algorithm [17], called “Propagation of Information” (PI). This initialization step produces a spanning tree with root at the sink. As we have already explained, each node lying on the spanning tree has a level, which is given by the level of its father plus one. In the case of the root, we say that it has a level equal to zero. The routing strategy that a given node follows is very simple; when the node decides to forward a packet to its father node, that is, along the spanning tree, we say that it uses a vertical routing; when it forwards a packet to any node of a level less or equal to that of its own, we say that it uses horizontal routing. It is very important to remark that, due to our modular design, a different settlement can be introduced to test alternative strategies.

#### 4.2.4. Congestion Control

The module in charge of congestion control is the core of this work. This module provides a self-organized and self-configurable congestion control distributed algorithm that works like a traffic light that controls the transit of packets on their way to the sink. Either let the packets to go forward or stop the packets at the buffers. The “green” and “red” lights depend on a function that intends to maximize the overall system satisfaction. The algorithm that rules each traffic light has been explained in the previous section.

#### 4.2.5. Wireless Effects

Our design considers the free space loss model, the effects of thermal noise, and interferences. We calculate the normalized signal-to-noise ratio and, for the binary phase-shift keying modulation (BPSK), we assess the bit error rate (BER). We determine the error probability per packet according to a fixed error tolerance. With this probability we are able to simulate packet losses due to wireless effects.

The simulations of wireless effects are considered and in the final statistics, it can be distinguished between packet losses due to wireless effects from those due to congestion.

The equation to calculate the signal power is

(11) $$\begin{array}{c} P_{r}=\frac{P_{t}\lambda^{2}}{{\left(4\pi d\right)}^{2}}, \end{array}$$

where *P*_*r*_ is the received signal power, *P*_*t*_ is the power of transmitted signal, *λ* is the wavelength, and *d* is the distance between transmitter and receiver.

Wireless simulation also includes the effects of thermal noise and interference in transmission of each node. We can calculate the maximum capacity of the wireless channel (*C*) using the Shannon capacity for a given bandwidth (*B*).

Using (12) we found the normalized signal-to-noise ratio (*E*_*b*_/*N*_*o*_). Consider

(12) $$\begin{array}{c} \frac{E_{b}}{N_{o}}=\frac{B}{C}\left(2^{C/B}-1\right). \end{array}$$

We have chosen BPSK because it requires less *E*_*b*_/*N*_*o*_ than other traditional modulation techniques. From a table [19] of BER for BPSK, BER is obtained, and considering the application requirements, specifically the bit error tolerance, the packet error rate (PER) is estimated as follows.

(13) $$\begin{array}{c} \text{PER}=1-\overset{e}{\underset{i=0}{\sum }}\left(\begin{array}{c} w\\ i \end{array}\right)\left[{\text{BER}}^{i}{\left(1-\text{BER}\right)}^{w-i}\right], \end{array}$$

where *e* is the number of bit errors tolerated by the application and *w* is the packet size in bits. The packet losses due to wireless effects are estimated by the PER.

#### 4.2.6. Energy Consumption

The WSN topology changes are represented using the power consumption simulation, due to the loss of connectivity caused by nodes that have depleted their battery energy. This effect is simulated by setting a percentage of consumed power in the data transmission. The consumption of energy due to processing capabilities is not considered in the simulation.

Packet losses resulting from the effects described in the previous paragraph are considered losses due to node level congestion. It is clear that as the network topology changes, an adaptive and self-configurable routing protocol is required to reduce node level congestion.

#### 4.2.7. Performance Evaluation

This module performs the recording of different parameters of the network, such as packet losses, offered traffic, throughput, and goodput. Different performance metrics are also calculated with the information obtained, for example, buffering time and lifetime of the network. Additionally, the user can propose other performance metrics, which can be drawn from the measurement of *ϕ*_*p*_ and *σ*_WSN_(*t*).

## 5. Results and Discussion

### 5.1. Simulation Results

In order to evaluate the benefits of using our congestion control scheme, four evaluation scenarios are proposed:

1. a scenario without congestion control,
2. an adaptive-routing scenario based on packet interference,
3. a medium access control based on CSMA and applying an adaptive-adjustment of back-off intervals,
4. a congestion control scenario.

The fourth scenario corresponds to our congestion control scheme, which also considers the use of mechanisms defined in 2 and 3, as previously explained in Section 3. Simulation settings for the abovementioned scenarios are shown in Table 2.

**Figure 2:**
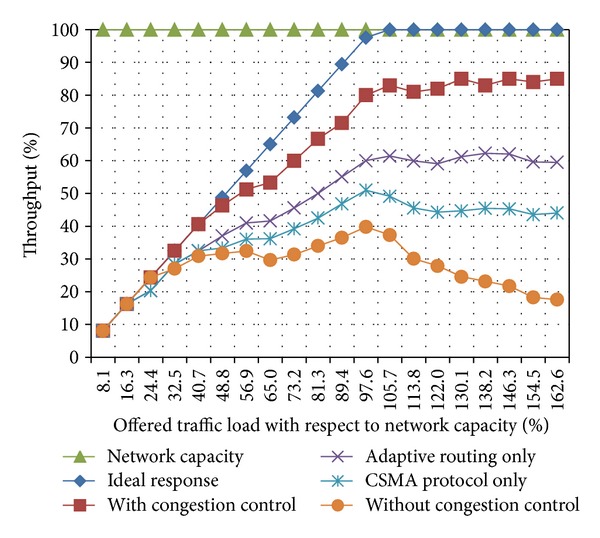
Throughput versus offered traffic load.

Figure 2 shows the performance of our congestion control scheme for the four proposed scenarios. From this figure, it can be observed that the WSN performance considerably decreases once generated traffic exceeds 65% of the WSN capacity. On one hand, if no congestion control mechanism is applied in this case, the WSN performance may be reduced up to a point where the network collapses. On the other hand, when our proposed congestion control is used, the amount of delivered packets approaches 80% of the WSN capacity. Figure 2 also illustrates the benefits of applying, combined and separated, both mechanisms considered in the proposed congestion control. It is worth pointing out that, due to the cross-layer approach used in the proposed congestion control mechanism, there is a synergistic effect while using both mechanisms.

**Figure 3:**
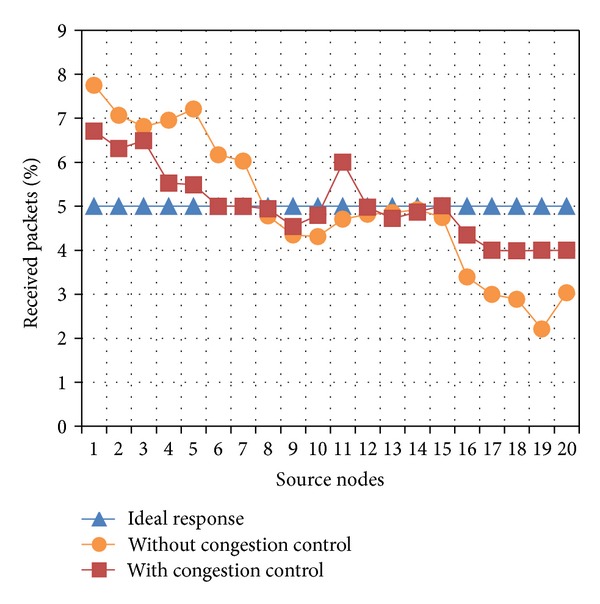
Percentage of received packets from each source node.

Another relevant advantage produced by the use of a congestion control mechanism is the equality of packet delivery. This goal is achieved upon adjusting specific congestion control parameters, which depend on node positions, in order to deliver information from all sources to sink node.  Figure 3 shows the performance of the proposed congestion control for a simulation scenario which consists of 1 to 20 source nodes. An* ID_number* is assigned to each source node; a small* ID_number* indicates that the corresponding source node is found closer to the sink node and vice versa. In this figure, it can be observed that the congestion control improves a fair packet delivery. Ideally, if *S* is the number of source nodes, for a fair packet delivery, the percentage of received packets would be given by *P* = 100/*S*.  Figure 3 shows the ideal percentage of received packets when the number of source nodes is *S* = 20 nodes; that is, *P* = 5%. As it can be observed from Figure 3, the use of the proposed congestion control increases the fairness in packet delivery.

In order to evaluate the fairness in packet delivery, we use Jain's Fairness Index [20]. In general terms, this metric provides a numerical estimate of how fair the resource assignment among network users is done by the system. In our case of study, we want to evaluate how fair the packet delivery from several sources to the sink in a WSN is performed. In order to use Jain's Fairness Index (JFI) to measure the average number of packets received by the sink from the source nodes, we use the following expression:

(14) $$\begin{array}{c} \text{JFI}=\frac{{\left(\sum_{j=1}^{m}{\hat{x}}_{j}\right)}^{2}}{m*\sum_{j=1}^{m}{\left({\hat{x}}_{j}\right)}^{2}}, \end{array}$$

where *m* corresponds to the number of source nodes in the network and ${\hat{x}}_{j}$ corresponds to the percentage of received packets from source *j*; (*x*_*j*_) is measured with respect to the percentage of expected packets (${\overset{-}{x}}_{j}$) from source *j*; that is,

(15) $$\begin{array}{c} {\hat{x}}_{j}=\frac{x_{j}}{{\overset{-}{x}}_{j}}. \end{array}$$

By using (14) with data presented in Figure 3, we obtained the following indexes:

 JFI = 88.81% (without using congestion control),
 JFI = 96.75% (using congestion control).

As it can be observed, the proposed control improves the fairness in packet delivery.

**Figure 4:**
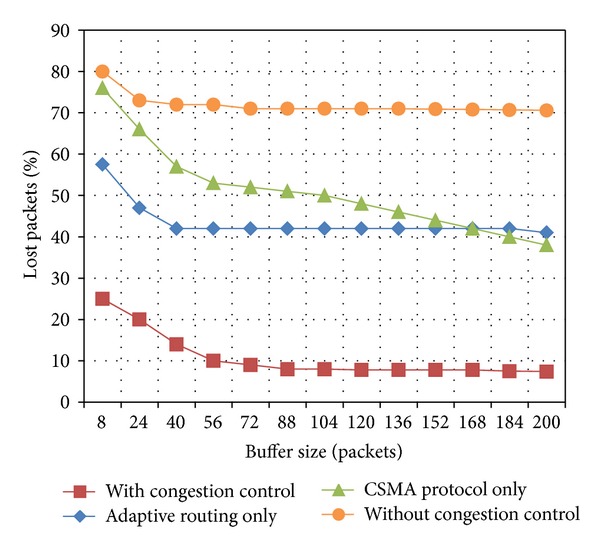
Lost packets versus buffer size for delay-tolerant applications.

**Figure 5:**
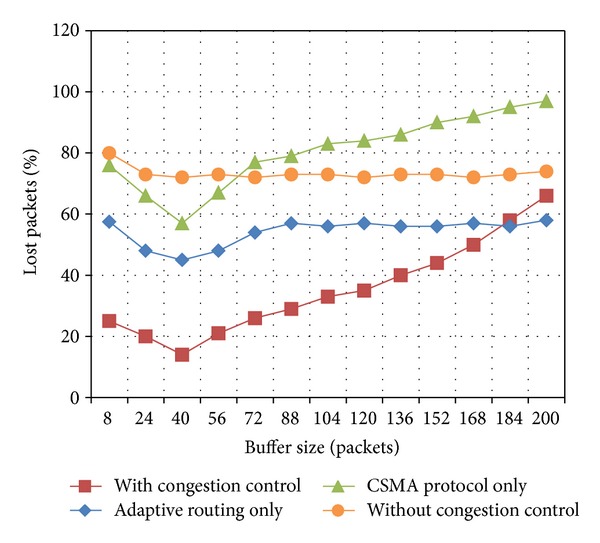
Lost packets versus buffer size for delay-sensitive applications.

As mentioned above, the number of packets in a flock depends on the buffer size. Hence, the buffer size is an important parameter for the proposed congestion control. In order to evaluate the impact of this parameter on the congestion control behavior, a series of simulations were conducted while changing the buffer size. Figures 4 and 5 illustrate the percentage of lost packets versus the buffer size while using the proposed congestion control and by individually using CSMA and adaptive routing as well as without using a congestion control mechanism.

In these experiments, we consider two case scenarios which depend on the WSN application, that is, delay-tolerant and delay-sensitive applications. For delay-tolerant applications, if the buffer size is increased, the packet drop rate decreases up to reaching a certain level when the full network capacity is achieved, as it can be observed in Figure 4. For delay-sensitive applications, generally a packet expiration time is established. This parameter limits the number of packets in a flock, in addition to the buffer size. As a consequence of such expiration time, even if the buffer size is increased, the packet loss may increase due to the fact that all packets arriving after this time will be discarded by the application, as it can be seen in Figure 5. For this case scenario, the percentage of lost packets comprises the percentage of dropped packets plus the percentage of packets arriving out of time to the sink.

**Figure 6:**
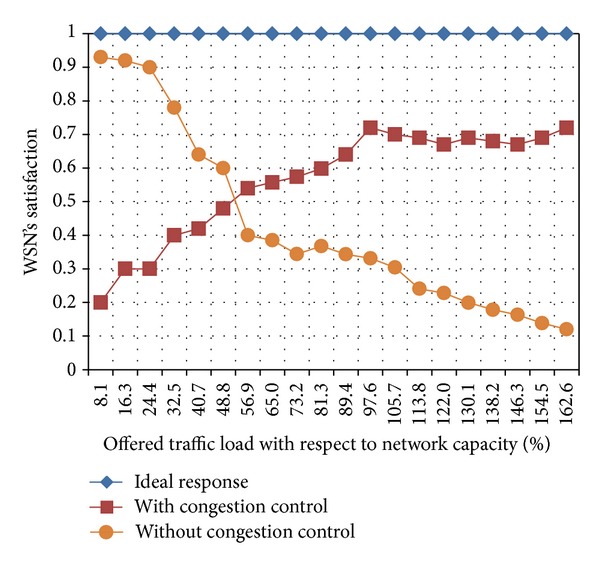
Network satisfaction as a function of the incoming traffic.

Figure 6 shows the overall network satisfaction for different values of offered load. For each possible value on the horizontal axis, we took a snapshot after the sink has started receiving the first packets, and therefore we can assume that the control mechanism is already acting at every node. The network satisfaction only considers those packets having an individual satisfaction above zero. Otherwise, packets with null or negative satisfaction will be dropped in the next possible moment. This figure also shows that our proposed control should be regarded as a reactive mechanism that is triggered when the offered load is greater than the 50% of the network capacity. Nevertheless, we get the best out of this control when the offered load is greater than the 100% of the network capacity.

The system requires some time to converge, and this time can be estimated as follows. Under these extreme conditions, it is considered that the offered load is over the full capacity of the network and flocks are assembled instantaneously. Then, the proposed adaptive mechanism reacts after the first packets arrive to the sink. Therefore, the time that the system takes to converge, that is, the time that the system takes to be in steady state, depends on the packet trip time, from the source(s) to the sink. If flocks are assembled with a certain delay, then the convergence will depend on the time it takes to assemble them, in addition to the packet trip time.

### 5.2. Discussion

The proposed congestion control provides a distributed solution. In this way, each node makes routing, contention, and selective packet dropping decisions based on its local traffic conditions. The main parameters of this solution are defined according to the zone where nodes are located, which can be translated on the hop distance between each node and the sink. Due to the fact that traffic conditions at each node of the WSN strongly depend on their distance to the sink node, traffic density is higher at zones closer to the sink node, and, therefore, it is expected that energy supply of these nodes is depleted more rapidly. The parameters of the congestion control, such as, packet lifetime, flock size, and maximum waiting time in the buffer node, are set up at each node according to its distance to the sink.

An important parameter of the proposed congestion control is given by the flock size, which must be adjusted with respect to buffer size. Flock size must be large enough to guarantee an effective operation of this solution. At this point, we can use as an analogy the vehicular traffic, where groups of vehicles are controlled by traffic lights. In this sense, a reduce-sized flock can be seen as a traffic light when the red light lasts only a short period of time, which is more suitable for a low traffic density. However, for a WSN with a high density of data packets, the size of the flock should be large; otherwise, it may endeavor collision avoidance and self-organization capabilities of the WSN. In contrast, extremely large-sized flocks may produce long transmission delays, because packets must remain in a buffer waiting for the arrival of the amount of packets settled by the flock size. Considering previous conditions, the flock size can be specified at node level according to the following two factors: (a) the packet density found in each zone of the WSN and (b) the packet arrival rate.

Due to the fact that the proposed method has been designed to work on scenarios with high traffic densities, we have decided to define the flock size according to the packet density, as observed in (8), where the flock size depends on node distance with respect to the sink node.

We consider that, for variants of WSN, such as query-based WSN, where traffic conditions fluctuate more frequently, it would be useful to establish other congestion control parameters taking into account, for example, the arrival packet rate.

The proposed solution is based on* packet overhearing*, because it uses a modified version of CSMA as the medium access control, and transmission requests are used among neighboring nodes.* Packet overhearing* may represent a disadvantage for applications that require a long-life WSN, beyond keeping a continuous packet transmission. However, for a high traffic density WSN, which is caused by the detection of an event of interest, the main purpose of the network is to inform about the evolution of this event, although this may imply that active nodes may deplete their energy supply soon.

We mention at the beginning of this work that an underlying assumption was that, as in economic systems (which are also complex systems), there is an “invisible hand” that guides individual packets to benefit the system through the pursuit of their private interests. Nevertheless, modern economy admits the government intervention to settle regulations that help agents to achieve synergy. We consider that this is the role of nodes within our theoretical framework. Thus, in our solution, each node decides the status of a packet, depending on its microscopic measures. These local decisions enforce congestion control as an emergent behavior at the system level.

### 5.3. Implementation Issues

About the overhead required to deploy the control mechanism that has been proposed, we focus the analysis on two aspects: (i) the amount of additional information that a packet should carry and (ii) the additional processing that a node must perform.

The packet overhead is minimum, because it is only the satisfaction value (*σ*_*p*,*M*_) achieved at the last node where the packet comes from.

As for the processing steps, each node has to calculate for each packet of a flock the following:

1. the local change of its satisfaction (Δ*σ*_*p*,*N*_, see (2) and (3)),
2. the accumulated satisfaction (*σ*_*p*,*N*_, see (4)),
3. the interference each packet induces on the rest of its flock (*ϕ*_*p*_, see (5)).

The first and second steps imply two subtractions, two divisions, and one addition, which means five elementary arithmetic operations. The third calculation seems to be expensive because (5) is invoked for each packet. Nevertheless, there is a simple shortcut that simplifies this step. Before the calculation of (*ϕ*_*p*_) we add all the changes calculated at Step (1). Let us call *X* to this result, which implies *F* − 1 additions. Then we enter into a loop to calculate each individual interference but this time we subtract Δ*σ*_*p*,*N*_ from *X*. We divide this result by (*F* − 1) and we have the second part of *ϕ*_*p*_. Finally, we again subtract this result from −Δ*σ*_*p*,*N*_. This step has taken three subtractions and one division, without considering the calculation of *X* (which is done only once). Besides this reduction in complexity we must recall that the calculation of *ϕ*_*p*_ is not always required. According to the forwarding procedure, *ϕ*_*p*_ is not necessary when none of the packets has experienced a reduction on its satisfaction. Otherwise, the node forwards immediately those packets whose satisfaction has been reduced. The node calculates the interference of each packet for the rest of the flock.

The number of packets that makes up a flock (*F*) plays a key role on the complexity of the processing steps that each node must perform. We also know that a flock occupies 1/3 of the buffer size for those nodes at zone 3 (the most distant places from the sink) and 2/3 of the buffers size for those nodes at zone 2, and it takes the overall buffer size for nodes in zone 1, which is in the vicinity of the sink. Therefore, the heaviest costs of this control are in charge of the nodes in zone 1. It is clear that these nodes define the carrying capacity of the network and therefore they are the busiest components of the system. This implies that these nodes may be the first to exhaust their energy budget. We consider that the deployed control contributes to extend the lifetime of these nodes, because there is a packet dropping mechanism acting at every node. This mechanism regulates the number of packets that finally arrive to the outskirts of the sink. Therefore, although the processing overhead mainly affects the nodes in zone 1, in compensation, the nodes in the other zones control the number of packets that arrive to zone 1. Also it is important to consider that, in terms of energy consumption, bit processing is less expensive than the corresponding transmission.

## 6. Conclusions

A conceptual framework for analyzing the traffic in wireless sensor networks (WSNs) from the “complex systems” perspective was developed. By means of this conceptual framework, it is possible to analyze the overall behavior of WSN traffic from local interactions of data packets. Although there is related work that analyzes the WSN as a “complex system,” the approach shown in our congestion control is different because the packets are considered as the main self-organized agents. In addition, the network congestion is mitigated using adaptive traffic mechanisms based on a satisfaction parameter assessed by each packet. These mechanisms have impact on the global satisfaction of the traffic carried by the WSN. Then the proposed congestion control is able to preserve the traffic stream close to the WSN capacity, even in case scenarios with extreme congestion.

For the developed control, packets are sent in groups, called flocks, assessing whether a packet is causing friction to the flock. The main idea of the proposed solution is to reduce friction among data packets by means of a synergic encouragement, which increases the overall performance. According to this idea, each node makes decision about routing, contention, and packet dropping in order to improve global performance. For instance, in specific scenarios, flocks are able to avoid congested zones of the WSN. The CSMA protocol is used for the medium access control, which is modified to consider the remaining space in the buffer node. As a consequence, nodes can decide if they are able to carry the packets and how many bits they can receive avoiding congestion not only at the link level, but also at the node level. In addition, nodes far from the sink keep their packets in buffer for shorter time than those packets near to the sink. As a result, the congestion control also improves fairness in packet delivery. Furthermore, our solution provides a novel and efficient way to improve the performance of WSN.

## Figures and Tables

**Table 1:** Complex systems concepts and their equivalences for the proposed WSN model.

Concept	WSN equivalent
Agent	Data packet
Goal	Arrive to the sink with minimum delay
Satisfaction^1^	0 if data packet does not arrive to the sink, 1 if it arrives to the sink within minimum time
Behavior	Packets travel across the network to the sink
Friction	An increase on the incoming traffic (throughput), reduces the outgoing traffic (goodput)
System's satisfaction	Can be evaluated from the outgoing traffic
Mediators	Nodes
Mediator's role	To execute a (distributed) algorithm that prevents congestion and maximizes the outgoing traffic

^1^Notice that satisfaction and friction are user-defined measures and they can change if required.

**Table 2:** Simulation settings.

Parameter	Value
Number of nodes	250
Number of source nodes	20
Packet size	2048 bits
Transmission rate	180 kb/s
Buffer size	16384 bits
Average degree of connectivity	6
Average coverage range	110 m
Percentage of consumed battery per transmission	0.01
Transmission frequency	2048 MHz
Transmission model	Free space loss
Modulation scheme	BPSK
Bandwidth	4 kHz
Generation packet rate	30 packets/s
